# Investigating the efficacy of dipotassium glycyrrhizin emollients in adult atopic dermatitis: A focused randomized parallel-controlled clinical study

**DOI:** 10.12669/pjms.41.7.9559

**Published:** 2025-07

**Authors:** Jiali Xia, Xinru Liu, Zixuan Wang, Yaxin Zhang, Guan Jiang

**Affiliations:** 1 Jiali Xia, Department of Dermatology, Affiliated Hospital of Xuzhou Medical University, Xuzhou, China; 2 Xinru Liu, Department of Dermatology, Affiliated Hospital of Xuzhou Medical University, Xuzhou, China; 3 Zixuan Wang, Department of Dermatology, Affiliated Hospital of Xuzhou Medical University, Xuzhou, China; 4 Yaxin Zhang, Department of Dermatology, Affiliated Hospital of Xuzhou Medical University, Xuzhou, China; 5 Guan Jiang, Department of Dermatology, Affiliated Hospital of Xuzhou Medical University, Xuzhou, China

**Keywords:** Atopic dermatitis, Clinical research, Dipotassium glycyrrhizin emollients, Emollients

## Abstract

**Background & Objective:**

Atopic dermatitis (AD), a chronic inflammatory skin condition, is primarily characterized by persistent itching and significant sleep disruption. Dipotassium glycyrrhizin, known for its anti-inflammatory properties, has shown potential in AD treatment. This study aims to evaluate the effectiveness of emollients containing dipotassium glycyrrhiza extract and the impact of varying application methods on improving the clinical severity of atopic dermatitis in adults during remission periods.

**Methods:**

systemic emollient, topical emollient, or no emollient, for an eight weeks duration. The study compared the incidence, disease assessment, and occurrence of adverse events across these groups.

**Results:**

After 12 weeks, the SCORAD score in the systemic emollient group decreased significantly by 6.56 points, more than the 4.03-point reduction in the topical emollient group (p < 0.05). The NRS itching score also showed notable decreases in both emollient groups compared to the no-emollient group (p < 0.05). The complete response rate was significantly higher in the emollient-treated groups than in the non-emollient group (p < 0.05).

**Conclusion:**

Regular use of dipotassium glycyrrhizin-containing emollients in adults with mild to moderate AD can effectively reduce symptoms and frequency of flare-ups, without notable adverse effects.

## INTRODUCTION

Atopic dermatitis (AD) is a chronic or episodic inflammatory skin disease, characterized by a cycle of dryness, intense itching, and eczematous lesions,^1^,^2^ alternating between acute exacerbations and stationary phases.^3^ This condition significantly impacts the quality of life, affecting about 10-30% of children and 2–10% of adults globally.^4^,^5^ The persistent nature of AD, with its frequent flare-ups, leads to considerable challenges for patients, including poor sleep, reduced self-esteem, and increased absenteeism. The persistent nature of AD, with its frequent flare-ups, leads to considerable challenges for patients, including poor sleep, reduced self-esteem, and increased absenteeism.^6^,^8^

Preventing the onset of AD episodes and reducing their severity during remission phases are vital for maintaining patient quality of life.^6^-^9^ Emollients^10^, as recommended by consensus-based guidelines, are integral to long-term AD management, focusing on hydrating the skin to alleviate dryness and itching. Despite the effectiveness of topical hormones like corticosteroids in AD treatment, their long-term safety concerns have led many patients to opt for moisturizers as a safer, sustained therapy alternative post the acute phase of AD.

In this evolving landscape of AD management, this study introduces an innovative approach by investigating the efficacy of moisturizers containing dipotassium glycyrrhizin, a compound with potential anti-inflammatory and immunomodulatory properties. The study aims to determine if these specialized moisturizers can sustainably alleviate the clinical severity of AD in adults during the maintenance phase. Moreover, it explores the comparative effectiveness of different application methods of these moisturizers, such as systemic versus topical application, providing insights into optimizing treatment strategies for AD. This research bridges a gap in understanding the role of novel ingredients in moisturizers and their practical application, potentially revolutionizing the approach towards long-term management of AD.

## METHODS

One hundred twenty patients with Atopic Dermatitis (AD) were selected from between October 2021 and February 2023. Informed consent was obtained from all participants.The moisturizer used in the study contained two primary ingredients: potassium glycyrrhizinate and allantoin.

### Ethical Approval:

The study was approved by the Ethics Committee of the Affiliated Hospital of Xuzhou Medical University (XYFY2022-KL 323-01, approved on October, 2021).

### Inclusion criteria:

- Patients meeting the Williams diagnostic criteria for AD with SCORAD scores under 50 points. Scores were classified as mild (0-25) and moderate (26-50).
- There were no gender restrictions, and the age range was 18-65 years.

### Exclusion criteria:

- Included acute AD with erosion, exudation, or secondary infection.
- Other active inflammatory skin diseases; recent use of antihistamines or topical drugs
- Recent treatments like phototherapy, systemic corticosteroids, or immunosuppressants.
- Known allergies to emollient ingredients or hydrocortisone cream.
- Participation in other clinical trials.
- Any conditions deemed unsuitable by the investigator.

### Methods

### Initial Treatment:

Patients received cetirizine hydrochloride tablets (10mg nightly) and butyrate cream applied once daily for two to four weeks.

### Maintenance Phase:

Patients with an Investigator Global Assessment (IGA) score of one entered the maintenance phase. Subjects were randomly divided into three groups:

### Follow-Up and Assessments:

Follow-ups occurred at weeks one, four, eight, and twelve. Parameters recorded included SCORAD score, Patient-Oriented Eczema Measure (POEM) score, Numerical Rating Scale (NRS) for itching, Atopic Dermatitis Control Tool (ADCT) score, and Dermatology Life Quality Index (DLQI) score. An episode was defined as an IGA score of two after seven days into the maintenance phase.

### Evaluation Index:

### Primary Outcomes:

Included SCORAD score assessment during remission at weeks one, four, eight, and twelve, along with the episode rate at the end of the eight weeks study period, median episode time, and duration from maintenance entry to episode onset.

### Secondary Outcomes:

Involved observation of SCORAD molecular components (pruritus and insomnia scores), DLQI, POEM score, NRS itching score, ADCT score, and objective sign score (assessing erythema, papules, exudation, scaling, and dryness). Safety was evaluated by recording adverse events (AEs).

### Statistical Analysis:

Data Representation: Count data were expressed as percentages (%) and compared using the chi-square test. Measurement data, following variance homogeneity and normal distribution, were represented as mean ± standard deviation.

### Comparative Analyses:

Group comparisons of episode rates were conducted using chi-square tests, with Fisher’s exact test for cases where chi-square was not applicable. The Wilcoxon Signed rank test was utilized for stratified data comparisons between groups. The Kaplan-Meier curve and log-rank test assessed episode onset times.

### Software and Significance:

Data were analyzed using SPSS version 26.0 (SPSS Inc., Chicago, IL, USA). A p-value of <0.05 was considered statistically significant.

## RESULTS

### Enrollment

The study involved 120 adult AD patients, comprising an equal number of men and women, aged between 18 and 65 years. During the trial, 45 patients were discharged due to AD attacks. Despite continued cetirizine and external medication, symptoms persisted, necessitating a revised treatment plan at the outpatient clinic. At week four, only 13 patients completed the treatment with topical emollient: three in Group-A (23.08%), two in Group-B (15.38%), and eight in Group-C (61.54%). By week 12, 16 additional patients were discharged.

### Clinical Outcomes:

### Baseline Measures:

The initial SCORAD scores ranged from 22.7 to 49.5 (mean 33.92 ± 7.60), including five patients with mild AD and 103 with moderate AD. There were no significant differences in gender and age among the groups, and baseline demographics and clinical characteristics were similar across all groups, as shown in Table-I.

**Table-I:** Basic patient data and baseline scores.

	A	B	C	P value
N	42	39	39	
Gender (male)	22 (52.4%)	21 (53.8%)	19 (48.7%)	0.897
Age (years)	34.5 (26.3)	36.0 (26.0)	41.0 (24.5)	0.578
***Pathogenic site (yes)***				
Head and neck	20 (47.6%)	17 (43.6%)	26 (66.7%)	0.092
Upper limbs	27 (64.3%)	22 (56.4%)	15 (38.5%) a*	0.060
Front and back	31 (73.8%)	20 (51.3%)	12 (30.8%) a***	<0.001
Lower limbs	26 (61.9%)	24 (61.5%)	14 (35.9%) a*, b*	0.029
Perineum	3 (7.1%)	1 (2.6%)	0 (0.0%)	0.191
***Signs and symptoms***				
Erythema	1.0 (2.0)	1.0 (2.0)	1.0 (2.0)	0.867
Edema	0.0 (0.8)	0.0 (0.0)	0.0 (1.0)	0.389
Exfoliation	0.0 (1.0)	1.0 (1.0)	1.0 (1.0)	0.353
Lichenification	0.0 (2.0)	0.0 (1.0)	1.0 (2.0)	0.594
Exudate and scab	0.0 (1.0)	0.0 (1.0)	0.0 (1.0)	0.335
Dry	2.0 (1.0)	2.0 (1.0)	2.0 (2.0)	0.949
Itch	5.0 (2.0)	5.0 (3.0)	5.0 (3.0)	0.539
Sleep	2.0 (2.0)	2.0 (3.0)	2.0 (3.0)	0.246
Recurrence (yes)	10 (23.8%)	15 (38.5%)	20 (51.3%) a*	0.038
Days of follow-up	91 (0.0)	91 (23.0)	91 (45.5) a**	0.016
***Baseline***				
Scorad	34.7 (13.1)	34.5 (13.0)	32.9 (12.4)	0.746
DLQI	7.0 (5.0)	8.0 (5.5)	6.0 (5.0)	0.211
POEM	9.0 (6.0)	10.0 (6.0)	10.0 (8.0)	0.480
NRS	5.5 (2.8)	5.0 (4.0)	5.0 (3.0)	0.862
ADCT	8.0 (4.8)	9.0 (6.5)	8.0 (6.0)	0.466

* Table-I Group statistical description, all using the median and interquartile spacing IQR Differences of four subgroups were tested by Kruskal-Wallis’ rank sum test. Post hoc was performed by Mann-Whitney U test with Bonferroni’s adjusted P values. The a and b represent the post hoc result with the group of ‘A’ and ‘B’, respectively, with * P < 0.05, **P < 0.01 and ***P < 0.001. Bonferroni Post-hoc test (corrected P-value, p’= p x 3 <0.05); Mann-Whitney U test is the Wilcox rank-sum test; Kruskal-Wallis’ rank sum test, is the P-value of the P value column; A * * *, b * * * is a pairwise Mann-Whitney U test or a chi-square test; a indicates difference from Group-A, b indicates difference from Group- B.

### SCORAD Scores:

**Fig.1:**
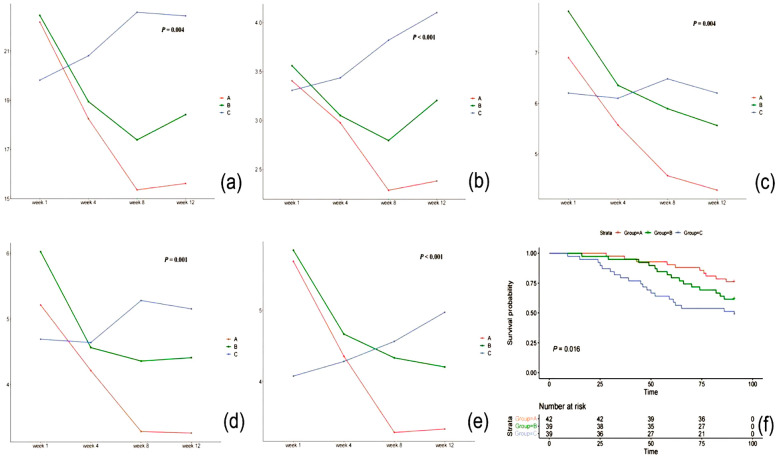
Plot of score changes during the maintenance period in the three groups.

At baseline, mean SCORAD scores in the three groups were similar (34.7, 34.5, and 32.9, respectively; P=0.746). After 12 weeks, both treatment groups (A and B) showed significant improvement in SCORAD scores compared to pre-treatment (P <0.05), as shown in Fig.1(a). The systemic emollient group (Group A) demonstrated a more significant decrease in SCORAD scores than the topical emollient group (Group B).as shown in Table-II.

**Table-II:** SCORAD, NRS, POEM, ADCT, DLQL score scale at weeks 1,4,8 and 12 of maintenance period of the three groups.

		A	B	C
Scorad	Week-1	22.18	22.45	19.82
	Week-4	18.25	18.95	20.81
	Week-8	15.36	17.39	22.58
	Week-12	15.62	18.42	22.43
NRS	Week-1	3.405	3.56	3.308
	Week-4	2.976	3.051	3.436
	Week-8	2.286	2.795	3.821
	Week-12	2.381	3.205	4.103
POEM	Week-1	6.905	7.821	6.205
	Week-4	5.571	6.359	6.103
	Week-8	4.571	5.897	6.487
	Week-12	4.286	5.564	6.205
ADCT	Week-1	5.214	6.026	4.692
	Week-4	4.214	4.564	4.641
	Week-8	3.286	4.359	5.282
	Week-12	3.262	4.41	5.154
DLQL	Week-1	5.69	5.846	4.077
	Week-4	4.357	4.667	4.282
	Week-8	3.286	4.333	4.564
	Week-12	3.333	4.205	4.974

### NRS Scores:

After 12 weeks, NRS scores decreased significantly in both treatment Group-s A and B, as shown in Fig.1(b), and increased in the no-emollient group (P <0.05), as shown in Table-II.

### POEM Scores:

Decreased continuously in treatment Groups- A and B, showing significant improvement at week 12 compared to baseline (P <0.05) in Fig.1(c). In the no-emollient group, the POEM score increased at week 8 and then returned to baseline at week 12 from as shown in Table-II.

### ADCT Scores:

Demonstrated significant improvement in treatment Groups-A and B after 12 weeks, with no substantial decrease in score from weeks 8 to 12 (P <0.05), shown in Fig.1 (d). The scores in the no-emollient group increased significantly between weeks 4 and 8 and decreased at week 12 were shown in Table-II.

### DLQI Scores:

Significantly lower in both treatment Groups-A and B after 12 weeks, whereas in the no-emollient group as shown in Fig.1(e), the DLQI score continuously increased during the maintenance period (P <0.05), as shown in Table-II.

### Comparative Analysis:

### Comparison at Week four:

At the four weeks mark of the maintenance period, the median POEM and DLQI scores were significantly lower in the systemic and topical emollient groups compared to the control group (P <0.05), as shown in Table-II.

### Atopic Dermatitis Episodes:

69.0% of patients treated with systemic emollients, 51.3% with topical emollients, and 35.9% of untreated patients experienced no episodes of atopic dermatitis. The attack rate was significantly lower, and the onset time longer in the treatment groups compared to the control group (P <0.05 or P <0.001), shown in Table-I.

### Onset Time Analysis:

### Median Onset Times:

For systemic treatment, it was 91 days (IQR 79.5-91), 91 days for the topical group (IQR 54-91), and 41 days for the control group (IQR 26-91). Survival analysis indicated that the onset time for Group-A was about 16.8% longer than Group-B, but not statistically significant (HR of Group-B / Group-A was 2.000; P=0.016), and significantly longer than Group-C, as shown in Fig.1(f).

## DISCUSSION

The study explores the clinical manifestation of Atopic Dermatitis (AD), a condition marked by acute or chronic pruritic skin lesions on dry skin, whose pathogenesis is linked to skin barrier defects, complex immune dysregulation, and IgE-mediated factors.^11^ Patients with moderate to severe AD often experience a diminished quality of life, grappling with chronic rash, severe itching, sleep deprivation, dietary restrictions, and psychosocial impacts. In this context, the efficacy of emollients, particularly in reducing dryness, emerges as a critical area of exploration.^12^ The treatment of dryness not only improves the quality of life for these patients^13^ but also reduces episodes and local corticosteroid depletion.^14^

The consensus for long-term AD management has typically revolved around maintaining disease stability post-remission, achieved through topical corticosteroids or calcineurin inhibitors.^10^ Emollients, by increasing skin hydration and combating dryness, play a pivotal role in repairing the skin barrier.^12^ This study innovatively extends this understanding by analyzing the adjunctive effects of emollients containing Dipotassium Glycyrrhizin in maintenance therapy for adult AD patients. It goes a step further by evaluating the impact of different application methods (systemic versus topical) on patient outcomes, thus providing new clinical insights into the effective application of emollients.

Dipotassium Glycyrrhizin (DG), a triterpene glycoside isolated from licorice root, is particularly noteworthy for its range of biological functions, including antitumor, antiviral, and anti-inflammatory effects. Its chemical stability, high solubility, and minimal side effects make it an ideal component for cosmetic and therapeutic applications.^15^ DG’s potential in AD treatment lies in its anti-inflammatory and immunomodulatory properties.^16^ Additionally, Allantoin (ALL), a compound promoting tissue repair and commonly found in skin care formulas, enhances the water absorption ability of skin and hair, aiding in the restoration of damaged skin.^17^

The study’s results demonstrate the significant impact of emollients containing Dipotassium Glycyrrhizin in reducing AD incidence and prolonging remission in adults. Systemic application, in particular, showed greater efficacy than topical application in reducing episodes and improving skin condition.^18^ Moreover, these emollients improved skin conditions by reducing pruritus and sleep disturbances and improved the quality of life, as reflected in lower DLQI scores.^19^

Comparatively, both systemic and topical use of emollients led to significant improvements in clinical measures, such as SCORAD index and DLQI score. The systemic application proved to be more effective in reducing episodes and improving skin condition, suggesting a preferable method of treatment in AD management.

This study offers significant advantages in AD management research. It examines the efficacy of dipotassium glycyrrhizin emollients in adult AD maintenance periods, addressing a literature gap. The comparison between systemic and topical application provides new clinical insights. Multiple assessment tools (SCORAD, POEM, NRS, ADCT, DLQI) were used to evaluate treatment outcomes comprehensively. Marked improvements in clinical indicators and quality of life demonstrate the intervention’s holistic benefits. The emollients showed good safety profiles while effectively reducing flare-up frequency and extending remission periods. The demonstrated superiority of systemic over topical application gives clinicians evidence-based guidance for treatment optimization.

### Limitations

This study has several limitations that should be considered when interpreting the results. First, the absence of a placebo control group using the same emollient base without dipotassium glycyrrhizinate limits our ability to attribute the observed effects specifically to dipotassium glycyrrhizinate rather than to the emollient base or psychological factors. Future studies should include such a placebo control arm to better isolate the specific contribution of the active ingredient. Second, the sample size was relatively small, and the study duration was limited to 12 weeks, which may not be sufficient to evaluate long-term efficacy and safety. Third, the study was conducted at a single center, potentially limiting the generalizability of the findings to different populations and clinical settings. Additionally, we did not measure inflammatory biomarkers, which would have provided objective evidence of the anti-inflammatory effects of the intervention. Despite limitations such as small sample size, short study duration, and lack of long-term follow-up, this study provides valuable insights into the effective use of dipotassium glycyrrhizinate-containing emollients in the maintenance treatment of adult AD. Future research should address these limitations and explore the effects of combining dipotassium glycyrrhizinate emollients with other treatment modalities.

## CONCLUSION

Our findings suggest that dermatologists should recommend regular whole-body application of emollients containing dipotassium glycyrrhizin during AD remission periods, as this approach significantly extends flare-free intervals and improves quality of life. This may reduce the need for corticosteroids and decrease healthcare utilization.

Despite limitations such as small sample size, short study duration, and lack of long-term follow-up, this study provides valuable insights into the effective use of dipotassium glycyrrhizinate-containing emollients in the maintenance treatment of adult AD. Future studies should expand the sample size and extend the study duration to obtain more reliable results.

### Authors’ contributions:

**JX** and **XL:** Conception and the design of the study.

**ZW, XL** and **YZ:** Acquisition, analysis and interpretation of the data. All authors have participated to drafting the manuscript.

**GJ:** Revised it critically.

All authors contributed equally to the study and read and approved the final version of the manuscript.
